# Reengineering of 7-dehydrocholesterol biosynthesis in *Saccharomyces cerevisiae* using combined pathway and organelle strategies

**DOI:** 10.3389/fmicb.2022.978074

**Published:** 2022-08-09

**Authors:** Wenqian Wei, Song Gao, Qiong Yi, Anjian Liu, Shiqin Yu, Jingwen Zhou

**Affiliations:** ^1^Engineering Research Center of Ministry of Education on Food Synthetic Biotechnology, and School of Biotechnology, Jiangnan University, Wuxi, China; ^2^Science Center for Future Foods, School of Biotechnology, Jiangnan University, Wuxi, China; ^3^Changsha Hospital for Maternal & Child Health Care, Changsha, China; ^4^Hunan Kerey Pharmaceutical Co., Ltd., Shaoyang, China; ^5^Jiangsu Provisional Research Center for Bioactive Product Processing Technology, Jiangnan University, Wuxi, China

**Keywords:** 7-dehydrocholesterol, “push and pull”, multi-copy site integration, metabolic engineering, *Saccharomyces cerevisiae*

## Abstract

7-Dehydrocholesterol (7-DHC) is a widely used sterol and a precursor of several costly steroidal drugs. In this study, 7-DHC biosynthesis pathway was constructed and modified in *Saccharomyces cerevisiae*. Firstly, the biosynthesis pathway was constructed by knocking out the competitive pathway genes *ERG5* and *ERG6* and integrating two *DHCR24* copies from *Gallus gallus* at both sites. Then, 7-DHC titer was improved by knocking out *MOT3*, which encoded a transcriptional repressor for the 7-DHC biosynthesis pathway. Next, by knocking out *NEM1* and *PAH1*, 7-DHC accumulation was improved, and genes upregulation was verified by quantitative PCR (qPCR). Additionally, *tHMG1*, *IDI1*, *ERG2*, *ERG3*, *DHCR24*, *POS5*, and *CTT1* integration into multi-copy sites was used to convert precursors to 7-DHC, and increase metabolic flux. Finally, qPCR confirmed the significant up-regulation of key genes transcriptional levels. In a 96 h shaker flask fermentation, the 7-DHC titer was 649.5 mg/L by *de novo* synthesis. In a 5 L bioreactor, the 7-DHC titer was 2.0 g/L, which was the highest 7-DHC titer reported to date. Our study is of great significance for the industrial production of 7-DHC and steroid development for medical settings.

## Introduction

7-Dehydrocholesterol (7-DHC) is a sterol found in animals, which can be directly converted to vitamin D3 after UV irradiation. Vitamin D3 is not only used to prevent and treat rickets and other bone diseases, but also improves immunity and reduces the cardiovascular disease risk (Guo et al., 2018). Vitamin D3 is widely used in biomedicine, feedstuffs, and other fields. 7-DHC is a precursor for 25-hydroxyvitamin D3 production, which is the active form of vitamin D3 and widely used to treat patients with severe liver and kidney disease (Warnke et al., 2016; Tang et al., 2020). Because current price of 25-hydroxyvitamin D3 is high and productivity is low, improving 7-DHC supplies is vital for steroids production.

Due to the wide applications and increasing demand for 7-DHC, its production in *Saccharomyces cerevisiae* is attractive. Su et al. (2015) ameliorated redox imbalance using a cofactor regeneration strategy and generated a titer of 44.49 mg/L (± 9.63 mg/L) in a 5 L bioreactor. Guo et al. (2018). increased precursor supply by overexpressing all mevalonate (MVA) pathway genes, and found the *DHCR24* from *Gallus gallus* was the most suitable gene to product 7-DHC in *S. cerevisiae*. Finally, the 7-DHC titer peaked at 1.07 g/L in a 5 L bioreactor. Guo et al. (2021) used the modular organelle localization of pathway genes to generate a 7-DHC titer of 360.6 mg/L in shaker flasks after 100 h. Qu et al. (2022) inhibited the expression of *ERG6* using CRISPRi, and the *S. cerevisiae* Ty1 transposon was used to increase the copies of key genes. The final 7-DHC titer was 365.5 mg/L in shaker flasks and 1,328 mg/L in a 3 L bioreactor.

**FIGURE 1:**
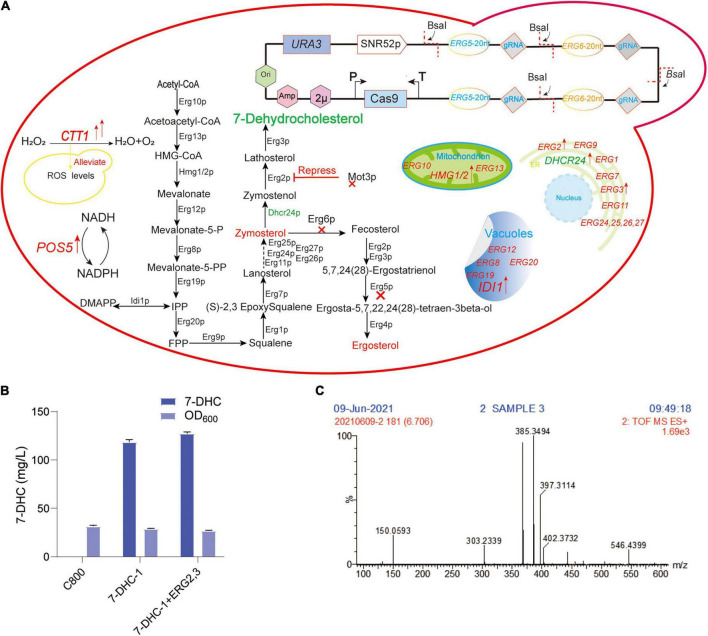
Preliminary 7-DHC synthesis in *S. cerevisiae.* **(A)** 7-DHC metabolic pathway in *S. cerevisiae*; **(B)** 7-DHC titer in preliminary synthesis in *S. cerevisiae*; **(C)** 7-DHC identification by LC-MS.

7-DHC is similar to ergosterol in *S. cerevisiae*, which is produced by ergosterol pathway modification, therefore *S. cerevisiae* is an excellent host for 7-DHC production (Shen et al., 2020; Xu and Li, 2020). Hmg1p (HMG-CoA reductase) and Idi1p (isoprenoid diphosphate isomerase) are the rate-limiting enzymes in the MVA pathway (Xia et al., 2022). Up-regulated *tHMG1* (truncated *HMG1*) and *IDI1* can increase squalene accumulation (Veen et al., 2003; Kwak et al., 2017). The endoplasmic reticulum (ER) is a critical organelle for 7-DHC biosynthesis (Hu et al., 2017; Jorda and Puig, 2020). Dhcr24p is the only exogenous enzyme of 7-DHC biosynthesis (Figure 1A). Mot3p is a transcriptional repressor of some genes under aerobic and hypoxia conditions (Hong et al., 2019) and directly inhibits *ERG2* and *ERG11* expression under hypoxia (Sertil et al., 2003; Montanes et al., 2011; Martinez-Montanes et al., 2013). Hongay et al. (2002) and Montanes et al. (2011) showed that Mot3p inhibited *ERG2* and *ERG9* transcription in *S. cerevisiae*. Under hypoxic conditions, *ERG2* mRNA levels were inhibited more than 10-fold. *PAH1* encodes a phosphatidic acid phosphatase, and its knockout improved ER-localized protein expression (Park et al., 2015; Arendt et al., 2017). Pah1p is dephosphorylated by the catalytic subunit (*NEM1*) of NEM1-SPO7 phosphatase (Su et al., 2014; Mirheydari et al., 2020), therefore, *NEM1* knockout may physically expand the ER. It can also expand the ER by overexpressing *INO2* (Carman and Han, 2009; Cirigliano et al., 2019; Kim et al., 2019). Upc2p and Ecm22p are involved in ergosterol regulation by binding to the *ERG* gene promoters (Davies et al., 2005; Yang et al., 2015; Joshua and Hofken, 2017). Iron is an important trace element for 7-DHC biosynthesis, e.g., the squalene monooxygenase Erg1p, the lanosterol C-14 demethylase Erg11p, the sterol C-4 methylase Erg25p, and the sterol C-5 desaturase Erg3p all require iron as a cofactor (Jorda et al., 2021).

Considering the current research status, the production capacity of *S. cerevisiae* must be improved to generate industrial 7-DHC levels for clinicians and patients. Due to its complex metabolic pathway, poor precursor conversion, and accumulation of intermediate sterols which impact on 7-DHC titer, driving pathway genes to convert most precursors to 7-DHC is required. *DHCR24* from *Gallus gallus* (Guo et al., 2018) was selected to generate 7-DHC. As *MOT3* inhibits *ERG2* expression, it was knocked out to enhance pathway gene expression. *NEM1* was also knocked out to enhance ER-localized gene expression. To reduce reactive oxygen species (ROS) in the cytoplasm, *CTT1* (cytoplasmic catalase) was overexpressed (Liu et al., 2021). Also, by enhancing *POS5* (mitochondrial NADH kinase) expression, NADPH supplies were increased. Finally, “push and pull” 7-DHC biosynthesis was achieved using the multi-copy site integration of *tHMG1*, *IDI*, *ERG2*, *ERG3*, *POS5*, *DHCR24*, and *CTT1*. The coordinated expression of all metabolic pathway genes was identified by qPCR. Finally, by *de novo* 7-DHC biosynthesis, the final 7-DHC titer was 649.5 mg/L (96 h) in shaker flasks, and 2.0 g/L in a 5 L bioreactor.

## Materials and methods

### Chemicals and standards

Standard 7-DHC was purchased from Shanghai Yuanye Co., Ltd., China, and ether and other chemicals were purchased from Shanghai Sinopharm Group, China. Enzymes for Golden Gate Assembly were purchased from NEB (Beijing).

### Strains, plasmids, and genes

*S. cerevisiae* strain C800 (CEN.PK2-1D; *MAT*α; *ura3-52*; *his3*Δ*1*; *trp1- 289*; *leu2-3*,*112*; *MAL2-8*^C^**; *SUC2*; *gal80*: *KanMX*) was used as the starting strain. *Escherichia coli* JM109 was used for plasmid construction and preservation. Heterologous, codon-optimized genes were synthesized by Sangon Biotech (Shanghai, China).

### Plasmid and strain construction

All fragments were cloned into a vector using the Gibson assembly kit (Gibson et al., 2009). Primers are listed in Supplementary Tables 1, 2. Engineered yeast was generated by episomal plasmid expression and genome integration. The yeast used in this study are listed in Supplementary Table 3.

This study used multiple gene editing technologies to knock out genes. Editor Benchling^1^ was used to design sgRNA, and the Golden Gate method (Apel et al., 2017) was used to construct multi-gene editing plasmids. The efficient *S. cerevisiae* transformation method was used for yeast transformations, and plasmids and fragments were transformed into yeast cells for expression and genome modification (Gietz and Woods, 2002).

### Multi-copy site integration

Histidine, tryptophan, and uracil tags were integrated with gene fragments, and relevant deficient yeast nitrogen base (YNB) culture medium was used to screen high-copy strains expressing integrated genes (Maury et al., 2016). Strains with the highest 7-DHC accumulation were screened in shaking flasks.

### Strain cultivation

Plasmid cloning and DNA extraction were performed using *E. coli* JM109 cells cultured in Luria-Bertani medium (10 g/L tryptone, 5 g/L yeast extract, and 10 g/L NaCl). Yeast expressing auxotrophic markers was selected on synthetic medium (20 g/L glucose, 1.74 g/L amino acid-free YNB, 5 g/L ammonium sulfate, 0.05 g/L leucine, 0.05 g/L histidine, 0.05 g/L tryptophan, and 0.05 g/L uracil). Engineered yeast strains were cultured in YPD (20 g/L glucose, 10 g/L yeast extract, and 20 g/L peptone). Selected colonies were cultured in YPD medium at 30°C and 220 rpm for 16–20 h, then an inoculum was added to 250 mL flasks containing 25 mL YPD medium. The initial OD_600_ was 0.2.

For 5 L fermentations, an engineered colony strain was inoculated into 15 mL YPD medium in a 250 mL shaker flask and cultured for 24 h at 220 rpm, 30°C. An inoculum (1%) was then transferred to a 250 mL flask containing 25 mL YPD medium (seed solution) and cultured for 17 h at 30°C and 220 rpm. Then, the culture was inoculated into a 5 L fermenter containing 2.5 L YPD medium at a 5% inoculum and a feed-batch fermentation initiated at 30°C, using 3 M NaOH to maintain the pH at 5.5. The feed medium (500 mL) was composed of: 400 g/L glucose, 18 g/L KH_2_PO_4_, 7g/L K_2_SO_4_, 0.56 g/L Na_2_SO_3_, 20 mL/L trace element A (comprising 5.75 g/L ZnSO_4_⋅7H_2_O, 0.32 g/L MnCl_2_⋅4H_2_O, 0.47 g/L CoCl_2_⋅6H_2_O, 0.48 g/L NaMoO_4_⋅2H_2_O, 2.9 g/L CaCl_2_⋅2H_2_O, 2.8 g/L FeSO_4_⋅7H_2_O, and 80 mL 0.5M EDTA, adjusted to pH 8.0), 24 mL/L trace element B (comprising 0.05 g/L biotin, 1 g/L calcium pantothenate, 1 g/L nicotinic acid, 25 g/L myo-inositol, 1 g/L thiamine HCl, 1 g/L pyridoxal HCl, and 0.02 g/L *p*-aminobenzoic acid), 1.0 g FeSO_4_⋅7H_2_O and 250 mL ethanol. The dissolved oxygen concentration was maintained at 25% by adjusting stirring rates (200–800 rpm). The feeding medium rate was 6–8 mL/h.

### Quantitative PCR

A fresh single yeast colony was inoculated into 5 mL YPD medium, cultured to the logarithmic growth phase for secondary transfer for 15–19 h, 1 mL cells aspirated and centrifuged, and a total RNA extraction kit (TaKaRa, Beijing, China) used to extract RNA. RNA was reverse transcribed to cDNA using a PrimeScript™ RT reagent kit with a gDNA eraser kit (TaKaRa). Quantitative PCR (qPCR) was conducted using the SYBR Premix Ex Taq II kit (TaKaRa). A LightCycler 480 II Real-Time PCR instrument (Roche Applied Science, Mannheim, Germany) was used for amplifications. *ACT1* functioned as an internal reference gene, and related gene transcription was determined using the 2^–ΔΔCt^ method (Chen et al., 2022).

### Processing and analysis methods

First, a potassium hydroxide ethanol saponification solution, with a mass fraction 30% (90% ethanol), was prepared. Next, 1 mL fermentation medium was centrifuged at 12,000 rpm for 8 min, the supernatant removed, resuspended in 1 mL saponification solution, and heated to 86–88°C for 3.5–4.0 h to undergo a reflux saponification reaction. Ether was the extractant (Wang et al., 2018). To a high performance liquid chromatography system (Waters, Milford MA, United States), a C18 column (30 m × 0.25 mm, 0.25 μm film thickness) was attached and maintained at 30°C. 7-DHC was separated in 100% methanol (mobile phase) at 280 nm at 1 mL/min in a separation time of 20 min (Xia et al., 2022). LC-MS method was used to identify 7-DHC (Sun et al., 2021).

## Results

### Constructing the 7-dehydrocholesterol pathway in *Saccharomyces cerevisiae*

In our previous study, the *S. cerevisiae* C800 host strain (CEN.PK2-1D; *MAT*α; *ura3-52*; *his3*Δ*1*; *trp1-289*; *leu2-3*,*112*; *MAL2-8*^C^**; *SUC2*; *gal80*:*KanMX*), which tolerated high glucose titer, was suitable for the *de novo* synthesis of exogenous sterols (Gao et al., 2020; Shi et al., 2021). To achieve *de novo* 7-DHC biosynthesis, *DHCR24* (heterologous Δ^24^-dehydrocholesterol reductase) was introduced to *S. cerevisiae*. In this study, two *DHCR24* copies were separately integrated into *ERG5* and *ERG6* sites, generating strain *7-DHC-1* (Figure 1A), which produced 118.5 mg/L 7-DHC in shaker flasks within 72 h (Figure 1B). *ERG2* and *ERG3* were overexpressed in *7-DHC-1* using the episomal expression vector pY26-TEF-GPD. However, this overexpression did not significantly increase the 7-DHC titer (Figure 1B). 7-DHC product was identified by liquid chromatography-mass spectroscopy (LC-MS) (Figure 1C).

### The effects of *MOT3* knockout on 7-dehydrocholesterol biosynthesis

**FIGURE 2:**
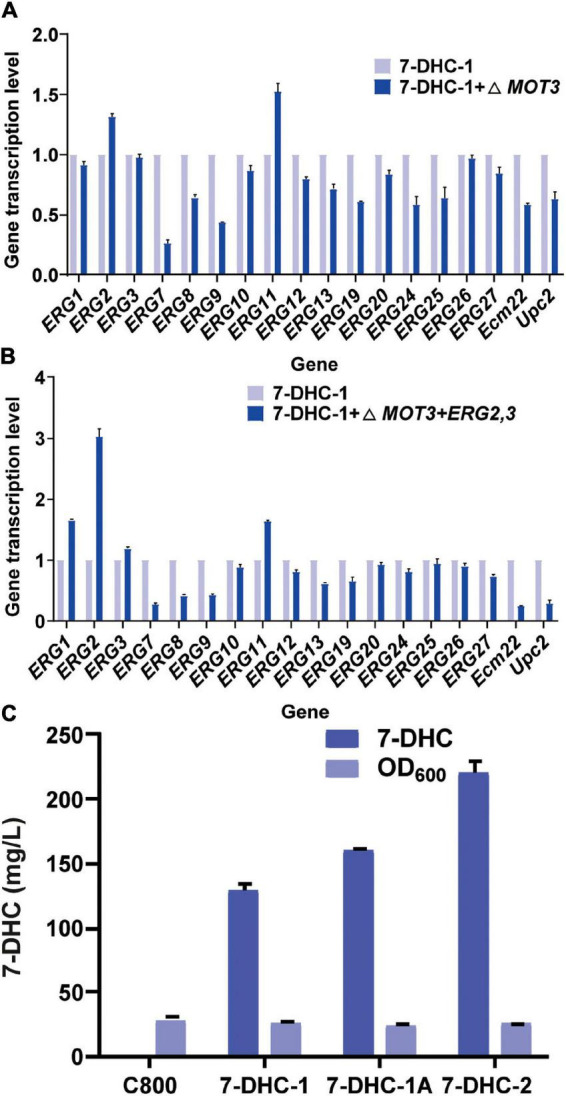
The knockout of related repressor genes in the 7-DHC synthesis pathway in *S. cerevisiae*. **(A)** qPCR validation of pathway genes after knockout of the hypoxia repressor *MOT3* in *S. cerevisiae*; **(B)** qPCR validation of pathway genes after one-copy integration of the *ERG2* and *ERG3* expression cassette at the *MOT3* site; **(C)** 7-DHC titers in *7-DHC-1*, *7-DHC-1A* (*7-DHC-1* + Δ*MOT3*), and *7-DHC-2* (*7-DHC-1* + Δ*MOT3* + *ERG2,3*) strains.

*ERG2* was previously identified as a key gene for 7-DHC and other sterol biosynthesis (Hongay et al., 2002). Similarly, some genes were found to inhibit *ERG2* expression (Hongay et al., 2002; Hong et al., 2019). For example, *MOT3*, which was identified as a transcriptional repressor for sterol biosynthesis pathways, inhibited *ERG2* expression under hypoxia. Thus, in this study, the knockout of *MOT3* in the *7-DHC-1* strain, could upregulate *ERG2* and *ERG11* transcription levels (Figure 2A), and could also increase the 7-DHC titer to 159.3 mg/L. This titer was increased by 24% when compared to the *7-DHC-1* strain. To further enhance precursor supplies for 7-DHC, one copy each of *ERG2* and *ERG3* were integrated at the *MOT3* site in the *7-DHC-1* strain, to generate the *7-DHC-2* strain. The qPCR results showed that *ERG2* transcriptional level was up-regulated, as were *ERG1*, *ERG3*, and *ERG11* levels (Figure 2B). The 7-DHC titer in the *7-DHC-2* strain was further increased from 128.7 mg/L to 220.2 mg/L within 72 h in shaker flasks (Figure 2C).

### Engineering organelle-related genes to enhance 7-dehydrocholesterol accumulation

**FIGURE 3:**
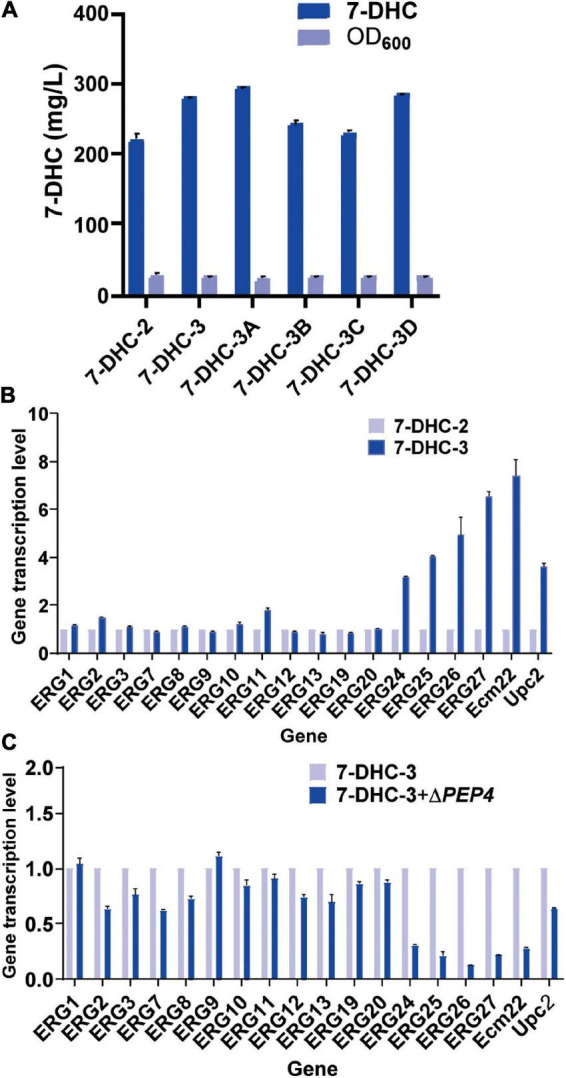
Engineering organelle related genes in *S. cerevisiae*. **(A)** 7-DHC titers in *7-DHC-2*, *7-DHC-3* (*7-DHC-2* + Δ*NEM1*), *7-DHC-3A* (*7-DHC-3* + Δ*PAH1*), *7-DHC-3B* (*7-DHC-3* + Δ*DGK1*), *7-DHC-3C* (*7-DHC-3* + Δ*PAH1* + Δ*DGK1*), *7-DHC-3D* (*7-DHC-3* + Δ*PEP4*) strains. **(B)** qPCR validation of pathway genes after *NEM1* knockout in *S. cerevisiae*. **(C)** qPCR validation of pathway genes after *PEP4* knockout in *S. cerevisiae*.

Previous studies reported that engineering cell organelles improved sterol accumulation (Arendt et al., 2017). Therefore, several genes related to ER and vacuole were engineered to evaluate their effects on 7-DHC accumulation. *NEM1* is reportedly responsible for *PAH1* dephosphorylation, which affects membrane areas of the ER and lipid metabolism. It was speculated that ER membrane expansion would enhance the expression of ER-localized genes. *NEM1* was knocked out in *7-DHC-2* to generate *7-DHC-3*, which produced up to 279.8 mg/L 7-DHC within 72 h in shaker flasks, and was 27.1% higher than *7-DHC-2* (Figure 3A). qPCR results showed that *NEM1* knockout enhanced the transcriptional levels of ER localization genes (Figure 3B). Furthermore, *PAH1* and *DGK1* knockout in *7-DHC-3* expanded the ER, and generated *7-DHC-3-1* (Δ*PAH1*), *7-DHC-3-2* (Δ*DGK1*), and *7-DHC-3-3* (Δ*PAH1* and Δ*DGK1*) strains. *PAH1* and *DGK1* knockout did not significantly increase 7-DHC accumulation, while the *DGK1* knockout reduced 7-DHC accumulation. The *PEP4* knockout in *7-DHC-3*, which was responsible for vacuole expansion and heterologous gene expression, generated strain *7-DHC-3-4*, which produced 284.4 mg/L 7-DHC and was slightly higher (9.3 mg/L) than *7-DHC-3*. However, qPCR results (Figure 3C) showed *PEP4* knockout caused transcriptional level downregulation of all pathway genes except *ERG9*. Thus, strain *7-DHC-3* was selected as the platform strain for further study.

### Multi-copy site integration coordinates gene expression

**FIGURE 4:**
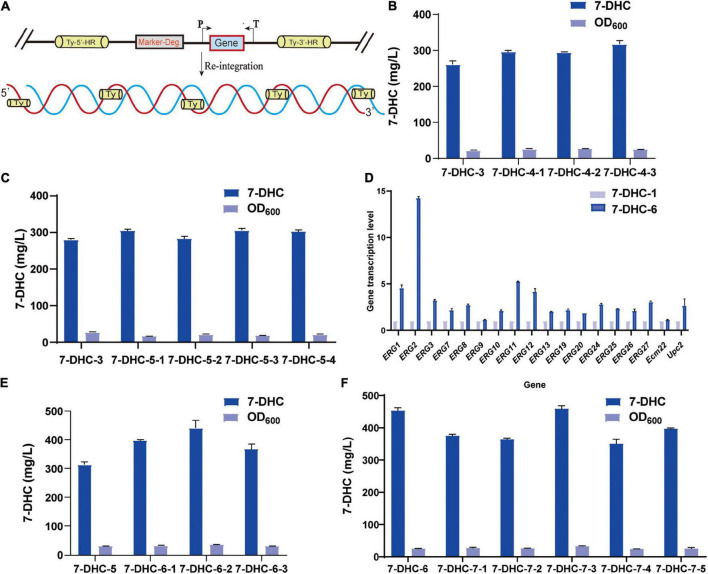
Multi-copy site integration coordinates gene expression. **(A)** Schematic showing multi-copy site integration. **(B)** Based on the *7-DHC-3* strain, the multi-copy integration of the rate-limiting genes *tHMG1* and *IDI* at the *Ty1* site was completed. **(C)** Based on the *7-DHC-3* strain, the multi-copy integration of *ERG2*, *ERG3*, *CTT1*, and *DHCR24* genes at the *Ty2* site was completed. **(D)** qPCR analyses of the *7-DHC-6-2* strain compared with the *7-DHC-1* strain. **(E)** Based on the *7-DHC-5* strain, the multi-copy integration of *tHMG1* and *IDI* at the *Ty1* site was completed. **(F)** Based on the *7-DHC-6* strain, the multi-copy integration of *ERG2*, *ERG3*, *POS5*, and *DHCR24* at the *Ty3* site was completed.

Based on *7-DHC-3*, *tHMG1* and *IDI* were integrated at the multi-copy site *Ty1*(Figure 4A). This *7-DHC-4* strain generated 56.0 mg/L more 7-DHC than *7-DHC-3* (Figure 4B); it produced 317.3 mg/L 7-DHC in shaker flasks within 72 h. Using *7-DHC-3*, *ERG2*, *ERG3*, *CTT1*, and *DHCR24* were integrated at the multi-copy site *Ty2*. This *7-DHC-5* strain produced 304.0 mg/L 7-DHC within 72 h in shaker flasks, which was 24.2 mg/L higher than *7-DHC-3* (Figure 4C). Using *7-DHC-5*, the rate-limiting genes *tHMG1* and *IDI* were integrated into *Ty1*. Finally, the *7-DHC-6* strain generated the highest 7-DHC titer in shaker flasks; 440.9 mg/L (72 h) which was 130.9 mg/L higher than *7-DHC-3* (Figure 4E). *7-DHC-6* analysis by qPCR confirmed that *ERG1*, *ERG2*, *ERG3*, *ERG7*, *ERG8*, *ERG9*, *ERG10*, *ERG11*, *ERG12*, *ERG13*, *ERG19*, *ERG20*, *ERG24*, *ERG25*, *ERG26*, *ERG27*, and transcriptional factors Ecm22p and Upc2p transcriptional levels were significantly higher than *7-DHC-1*. Of these genes, *ERG2* was up-regulated 14-fold (Figure 4D). Based on *7-DHC-6*, *ERG2*, *ERG3*, *POS5*, and *DHCR24* were integrated into *Ty3*. This *7-DHC-7* strain generated a 7-DHC titer of 459.3 mg/L within 72 h in shaker flasks, which was 18.4 mg/L higher than *7-DHC-6* (Figure 4F).

### Fermentation optimization of the 7-dehydrocholesterol high-yielding strain

**FIGURE 5:**
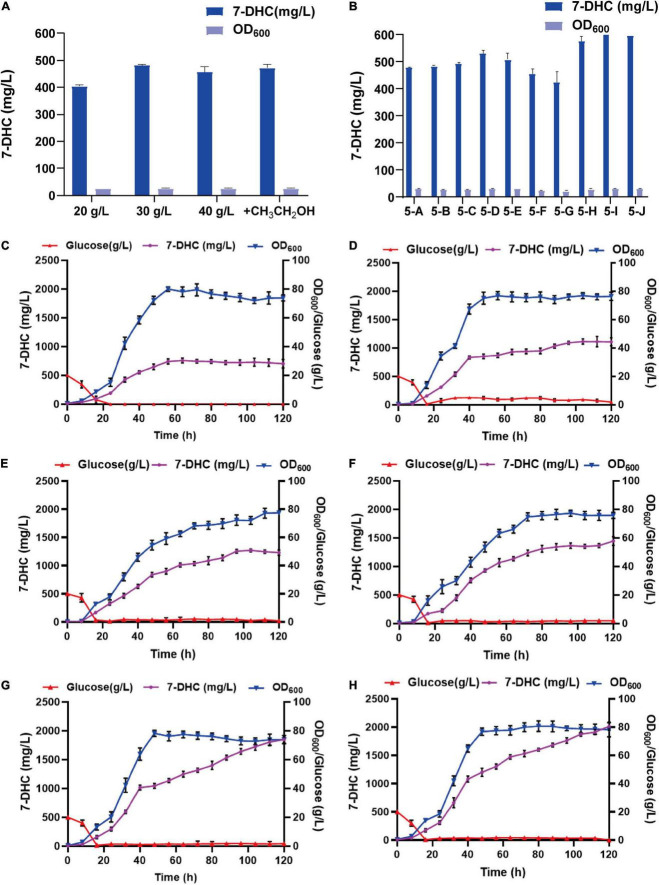
Optimizing 7-DHC production in *S. cerevisiae*. **(A)** The effects of glucose and ethanol (20 g/L) on 7-DHC accumulation. The initial concentration of glucose is controlled as 20 g/L, 30 g/L, 40 g/L. Ethanol was added at the 24th hour of fermentation, based on the initial glucose concentration of 20 g/L. **(B)** The effects of Mg^2+^ and Fe^2+^ on 7-DHC accumulation. 7-DHC titers in 5-A (20 g/L glucose), 5-B (20 g/L glucose + 0.05 g/L Fe^2+^), 5-C (20 g/L glucose + 0.1 g/L Fe^2+^), 5-D (20 g/L glucose + 0.3 g/L Fe^2+^), 5-E (20 g/L glucose + 0.5 g/L Fe^2+^), 5-F (20 g/L glucose + 1 g/L Mg^2+^), 5-G (20 g/L glucose + 3 g/L Mg^2+^), 5-H (30 g/L glucose + 0.3 g/L Fe^2+^), 5-I (30 g/L glucose + 0.4 g/L Fe^2+^), 5-J (30 g/L glucose + 0.5 g/L Fe^2+^) strains. **(C)** 7-DHC production by feed-batch fermentation in a 5 L bioreactor, and the dissolved oxygen concentration was maintained at 40%. **(D)** 7-DHC production by feed-batch fermentation in a 5 L bioreactor. The dissolved oxygen concentration was maintained at 30% and glucose concentration was maintained at 3–4 g/L. **(E)** 7-DHC production by feed-batch fermentation in a 5 L bioreactor. The dissolved oxygen concentration was maintained at 30% and glucose concentration was maintained at 0–2 g/L. **(F)** 7-DHC production by feed-batch fermentation in a 5 L bioreactor. The dissolved oxygen concentration was maintained at 30–10% and glucose concentration was maintained at 0–2 g/L (add ethanol after 72 h). **(G)** 7-DHC production by feed-batch fermentation in a 5 L bioreactor. The dissolved oxygen concentration was maintained at 25% and glucose concentration was maintained at 0–2 g/L (add ethanol after 72 h). 0.4 g/L FeSO_4_⋅7H_2_O was added at the time of inoculation. **(H)** FeSO_4_⋅7H_2_O was added by fed-feed method to the final concentration of 0.4 g/L.

By analyzing the 7-DHC production metabolic pathway in *S. cerevisiae*, the effects of carbon sources and trace elements were explored on 7-DHC accumulation. Also, by examining the exogenous addition of the carbon sources, glucose and ethanol (Figure 5A), and the trace elements, Mg^2+^ and Fe^2+^ (Figure 5B), 7-DHC accumulation peaked when initial glucose was 30 g/L and ferrous sulfate was 0.4 g/L (Figure 5B). 7-DHC accumulation was 649.5 mg/L within 96 h in shaker flasks, and 2.0 g/L (Figures 5C–H) in a 5 L bioreactor.

## Discussion

In this study, the combinatorial engineering strategies were used to construct several 7-DHC producing strains. Exogenous *DHCR24* was introduced, then, the off-pathway genes *MOT3* and *NEM1* were knocked out, the rate-limiting step genes *tHMG1* and *IDI* were overexpressed to increase precursor supply, and *ERG2*, *ERG3*, *DHCR24* expression was enhanced to convert precursors to 7-DHC. The multi-copy integration of the cytoplasmic catalase gene *CTT1* was used to reduce cytoplasmic ROS levels. The *POS5* was overexpressed to increase NADPH supply. Finally, *de novo* 7-DHC synthesis was achieved in *S. cerevisiae*; the 7-DHC titer was 649.5 mg/L within 96 h in shaker flasks and 2.0 g/L at 120 h in a 5 L bioreactor.

From previous research, enhancing ER-localized *ERG2* and *ERG3* expression had no significant effects on 7-DHC accumulation (Guo et al., 2021). However, *ERG2* and *ERG3* are both key genes for 7-DHC accumulation. *MOT3* inhibited *ERG2* expression under hypoxic conditions, therefore, in this study, *ERG2* and *ERG3* were integrated into *MOT3* site, which significantly increased the 7-DHC titer. Hongay et al. (2002) showed that *S. cerevisiae* experienced a > 10-fold inhibition of *ERG2* mRNA levels under hypoxic conditions. In *MOT3* deletion mutants, *ERG2* inhibition was almost completely eliminated under hypoxia. Also, *ERG1*, *ERG3*, and *ERG11* expression were somewhat up-regulated. Since *ERG1*, *ERG2*, *ERG3*, and *ERG11* are key rate-limiting genes in the 7-DHC biosynthesis pathway, the *MOT3* knockout benefitted their expression (Sertil et al., 2003; Montanes et al., 2011; Martinez-Montanes et al., 2013).

Increasing ER membrane area benefits the expression of most genes in the 7-DHC biosynthesis pathway. *PAH1* knockout reportedly dilated the ER, while *NEM1* was responsible for *PAH1* dephosphorylation, thereby affecting ER membrane area and lipid metabolism (Carman and Han, 2009; Arendt et al., 2017; Kim et al., 2019). According to Park et al. (2015), *DGK1* knockout alleviated the effects of *PAH1* knockout on yeast growth. *PEP4* encodes a protease, which was shown to mature different vacuolar peptidases; however, the *PEP4* knockout positively affected vacuole expansion and heterologous gene expression (Arendt et al., 2017). In this study, after *NEM1* was knocked out, the 7-DHC titer increased by 27.1%. Based on this knockout, the 7-DHC titer increased when *PAH1* was knocked out, but cell optical density decreased. However, the 7-DHC titer decreased after both *DGK1* and *PAH1* were knocked out. Therefore, *NEM1, PAH1*, and *DGK1* could not be simultaneously knocked out, as lipid metabolism was seriously affected. After the *PEP4* knockout, most 7-DHC pathway genes except *ERG9* were down-regulated.

In *S. cerevisiae*, Upc2p and Ecm22p are similar zinc-finger transcriptional factors that activate transcription by binding to *ERG* promoters involved in ergosterol biosynthesis and uptake. The 888^th^ gene mutation of Upc2p and the 790th of Ecm22p both increased transcriptional activation (Davies et al., 2005; Yang et al., 2015). The C-termini of Upc2p and Ecm22p are responsible for ergosterol binding; however, it is unclear if other sterols in *S. cerevisiae* bind to transcription factor C-termini. Therefore, the effects of two different C-terminal truncated forms of the transcription factor Ecm22p were explored on 7-DHC accumulation. When two Ecm22p forms, Ecm22-1 (1,500 bp) and Ecm22-2 (1,200 bp), were overexpressed, their 7-DHC titers were unchanged in both forms.

As many genes are involved in 7-DHC biosynthesis in *S. cerevisiae*, the conversion efficiency of precursors directly leads to limited 7-DHC accumulation. Therefore, a strategy was used to enhance the accumulation of 7-DHC by increasing precursor conversion. Firstly, the multi-copy integration of *tHMG1* and *IDI* at *Ty1* was engineered to enhance downstream acetyl-CoA metabolism. Also, 7-DHC accumulation was increased after the multi-copy integration of *ERG2*, *ERG3*, *CTT1*, and *DHCR24* at *Ty2*. Finally, the 7-DHC titer at 72 h in shaker flasks was 440.9 mg/L, which was 130.9 mg/L higher than in control strain. This strategy benefitted 7-DHC accumulation in *S. cerevisiae.* By verifying the *7-DHC-6* strain (qPCR), *ERG2* transcriptional levels were increased 14-fold and *ERG11* fivefold. *MOT3* knockout alleviated metabolic inhibitory effects and significantly promoted the expression of key genes were verified. Finally, *POS5* expression was enhanced to further increase 7-DHC titers and cell density. By exploring carbon sources and trace element addition, it was observed that 7-DHC accumulation peaked when the initial glucose concentration was 30 g/L and the initial ferrous sulfate was 0.4 g/L.

## Conclusion

In conclusion, this study successfully reengineered the 7-DHC biosynthesis pathway in *S. cerevisiae*, and used metabolic strategies, including related genes knockout, intracellular ROS reduction, and enhanced precursor supply and conversion, to improve the 7-DHC production. Finally, the 7-DHC titer was 649.5 mg/L in shaker flasks and 2.0 g/L in a 5 L bioreactor. This study lays the foundations for the industrial production of 7-DHC, and provide a platform for the successful production of other steroids in *S. cerevisiae*.

## Data availability statement

The original contributions presented in the study are included in the article/Supplementary material, further inquiries can be directed to the corresponding author/s.

## Author contributions

WW: experimental design and implementation, date analysis, and writing. SG: experimental design. QY and AL: data analysis. SY and JZ: reviewing and editing. All authors contributed to the article and approved the submitted version.

## Footnotes

1
https://benchling.com/editor
