# Supplementary material for: Reengineering of 7-dehydrocholesterol biosynthesis in Saccharomyces cerevisiae using combined pathway and organelle strategies
Source: Front Microbiol. 2022 Aug 9;13:978074. doi: 10.3389/fmicb.2022.978074 (PMC9398459; doi:10.3389/fmicb.2022.978074)
Supplement: Supplementary file 1 [file Data_Sheet_1.docx]

Supplementary Material

# Supplementary Tables

**Table S1 the primers of the metabolic pathway construction and gene knockout**

| **Oligonucleotides** | **Sequences (5'-3')** |
| --- | --- |
| MOT3-UP-F- PRS424 | CACTAAAGGGAACAAAAGCTGCCAAGGCGCATCATCCTGTC |
| MOT3-UP-R | ACACAACTAAAAGCAATTACAGTCAGCGAATCGCTGCCGATATTG |
| PGK1P- F- MOT3 | CGGCAGCGATTCGCTGACTGTAATTGCTTTTAGTTGTG |
| PGK1P-R-ERG2 | AAACTTCATTGTTTTATATTTGTTGTAAAAAGTAGATAATTACTTCCTTGATGA |
| ERG2-F-PGK1P | CTTTTTACAACAAATATAAAACAATGAAGTTTTTCCCACTCCTTTTGTTGATTG |
| ERG2-R-TEFT | CGGCCGGTACCTTAGAACTTTTTGTTTTGCAACAAGTTCTTACCC |
| TEFT-F-ERG2 | GTTGCAAAACAAAAAGTTCTAAGGTACCGGCCGCAAATTAAAGC |
| TEFT-R-ERG3 | CAAGAAGAACAACTGACTCGAGTCATGTAATTAGTTATGTCACGCTTAC |
| ERG3-F-TEFT | ACTAATTACATGACTCGAGTCAGTTGTTCTTCTTGGTATTTGGGTCG |
| ERG3-R-TDH3P | CACACATAAACAAACAAAATGGATTTGGTCTTAGAAGTCGCTG |
| TDH3P-F-ERG3 | CTAAGACCAAATCCATTTTGTTTGTTTATGTGTGTTTATTCGAAACTAAGTTC |
| TDH3P-R-MOT3 | GTTATTCAAAACACGATAAAAAACACGCTTTTTCAGTTCGAGTTTATC |
| MOT3-DOWN-F-TDH3P | AAGCGTGTTTTTTATCGTGTTTTGAATAACCTTATTGTTAGTCACAACA |
| MOT3-DOWN-R-PRS424 | GGGCGAATTGGATAATTTCATTGAATTCATCAAGAGATTTGAAACAAA |
| PRS424-MOT3-DOWN-F | CTTGATGAATTCAATGAAATTATCCAATTCGCCCTATAGTGAGTCGT |
| PRS424-MOT3-UP-R | GATGATGCGCCTTGGCAGCTTTTGTTCCCTTTAGTGAGGG |
| 5-1-MOT3-PAM-1-F | AAAGGTCTCTGATCCACAACAACCTCAACAATGGGTTTTAGAGCTAGAAATAGCAAGTTAAAATAAGGC |
| 5-1-MOT3-PAM-1-R | TTTGGTCTCTCAGGTGCGCAAGCCCGGAATCG |
| 5-2-MOT3-PAM-2-F | AAAGGTCTCTCCTGGTTGGAAAATAAACACGTTTTAGAGCTAGAAATAGCAAGTTAAAATAAGGC |
| 5-2-MOT3-PAM-2-R | TTTGGTCTCTAGTGTGCGCAAGCCCGGAATCG |
| 5-3-MOT3-PAM-3-F | AAAGGTCTCTCACTGCGAATATGCCAATACGTTTTAGAGCTAGAAATAGCAAGTTAAAATAAGGC |
| 5-3-MOT3-PAM-3-R | TTTGGTCTCTGTCGTGCGCAAGCCCGGAATCG |
| 5-4-MOT3-PAM-4-F | AAAGGTCTCTCGACGTTATGGTGAACTCTGGTTTTAGAGCTAGAAATAGCAAGTTAAAATAAGGC |
| 4-1-ERG5-PAM-1-F | AAAGGTCTCTGATCATTTCATGGAAAAAGACCTGGTTTTAGAGCTAGAAATAGCAAGTTAAAATAAGGC |
| 4-1-ERG5-PAM-1-R | TTTGGTCTCTAAGATGCGCAAGCCCGGAATCG |
| 4-2-ERG5-PAM-2-F | AAAGGTCTCTTCTTAGCTAAGACATCTGGAGTTTTAGAGCTAGAAATAGCAAGTTAAAATAAGGC |
| 4-2-ERG5-PAM-2-R | TTTGGTCTCTGACGTGCGCAAGCCCGGAATCG |
| 4-3-ERG6-PAM-1-F | AAAGGTCTCTCGTCGACATGGAACATCTTTGTTTTAGAGCTAGAAATAGCAAGTTAAAATAAGGC |
| 4-3-ERG6-PAM-1-R | TTTGGTCTCTCTGGTGCGCAAGCCCGGAATCG |
| 4-4-ERG6-PAM-2-F | AAAGGTCTCTCCAGGTCTTCGCTAACGAGGGTTTTAGAGCTAGAAATAGCAAGTTAAAATAAGGC |
| ERG5-UP-F | GTGAGTATTTGTTTGTAAAGTCGCACCTTTAG |
| ERG5-UP-R-TEFP | TTTGAAGCTATGAGCTCCAAAGAAGAATTGGTTGGTTTTTGGTTGT |
| TEFP-ERG5-F | CAATTCTTCTTTGGAGCTCATAGCTTCAAAATGTTTCTACTCC |
| TEFP-DHCR24-R | CCAAACAGCAGACATACTAGTTCTAGAAAACTTAGATTAGATTGCTATGC |
| DHCR24-F-TEFP | CTAAGTTTTCTAGAACTAGTATGTCTGCTGTTTGGTCTTTAGGTG |
| DHCR24-R-TEFT | CATGACTCGAGTTAATGTCTTGCTGCTTTACAAATTTTATCATAAACTTC |
| TEFT-DHCR24-F | TTTGTAAAGCAGCAAGACATTAACTCGAGTCATGTAATTAGTTATGTCACGC |
| TEFT-ERG5-DOWN-R | AATATTTGCTACTTTAATCTGTATTCTGGTACCGGCCGCAAATTAAAGC |
| ERG5-DOWN-F-TEFT | GTACCAGAATACAGATTAAAGTAGCAAATATTTTCAAATATGACATAG |
| ERG5-DOWN-R | GCCGGTGTTTATATCGCTATTGAAGAGAGC |
| ERG6-UP-F | GCTTTATAACACTATTCTTAGATTTCACTTTTACCATATCG |
| ERG6-UP-R-TEFP | GAAACATTTTGAAGCTATGAGCTCCTGCGGTTGGTTTAGTTGCCGGT |
| TEFP-ERG6-UP-F | CCAACCGCAGGAGCTCATAGCTTCAAAATGTTTCTACTCC |
| TEFT-ERG6-DOWN-R | CCCAAAAGGAAGCCGTTCAGAGGTACCGGCCGCAAATTAAAGC |
| ERG6-DOWN-R | GTGAGGAGCAGACAATGTATTATTCACTGTAAAC |
| 7-3-NEM1-PAM-1-F | AAAGGTCTCTCACACCGATAAGAGAAACAGGTTTTAGAGCTAGAAATAGCAAGTTAAAATAAGGC |
| 7-3-NEM1-PAM-1-R | TTTGGTCTCTCTTCTGCGCAAGCCCGGAATCG |
| 7-4-NEM1-PAM-2-F | AAAGGTCTCTGAAGTGAAGTTTGGATTGAGGTTTTAGAGCTAGAAATAGCAAGTTAAAATAAGGC |
| 1-4-R | TTTGGTCTCTAAAATAAATTGGCCATAGAAAAATTCTGTTATCCAC |
| NEM1-UP-F | CGTAAATTCTGTTTCCGAAGATGCAAG |
| NEM1-UP-R | GTTGTTAATTTCTGGACATTGTTTCATTAATTGA |
| NEM1-DOWN-F | CAATTGTTATTGTGTCCTTCCAAAAAAGATG |
| NEM1-DOWN-R | GTTCGTTCTGCTATCCTAACTAATTGCAG |
| 8-1-PEP4-PAM-1-F | AAAGGTCTCTGATCAGAAGTACCAGTATCGATGGGTTTTAGAGCTAGAAATAGCAAGTTAAAATAAGGC |
| 8-1-PEP4-PAM-1-R | TTTGGTCTCTATGGTGCGCAAGCCCGGAATCG |
| 8-2-PEP4-PAM-2-F | AAAGGTCTCTCCATTTTCAGTATCCTTTGAGTTTTAGAGCTAGAAATAGCAAGTTAAAATAAGGC |
| 8-2-PEP4-PAM-2-R | TTTGGTCTCTGCTGTGCGCAAGCCCGGAATCG |
| 8-3-PEP4-PAM-3-F | AAAGGTCTCTCAGCGAAGTCTTGTTTTGGAGTTTTAGAGCTAGAAATAGCAAGTTAAAATAAGGC |
| 8-3-PEP4-PAM-3-R | TTTGGTCTCTCAACTGCGCAAGCCCGGAATCG |
| 8-4-PEP4-PAM-4-F | AAAGGTCTCTGTTGGCGCTGACCAACAACAGTTTTAGAGCTAGAAATAGCAAGTTAAAATAAGGC |
| PEP4-UP-F | GAACCAGAACTAGACCTCGTTGATGG |
| PEP4-UP-R | TTCAAACAAAAACCAAAACTAACGCTAAACTTTTCTTACTTCTCCGCCC |
| PEP4-DOWN-F | GAAAAGTTTAGCGTTAGTTTTGGTTTTTGTTTGAATTTTATTTGGATT |
| PEP4-DOWN-R | GCTGAGGTCTGATTATTTCTATAACCAAAAGC |
| 9-1-DGK1-PAM1-F | AAAGGTCTCTGATCGGAGATTCATACATACAATGGTTTTAGAGCTAGAAATAGCAAGTTAAAATAAGGC |
| 9-1-DGK1-PAM1-R | TTTGGTCTCTTACGTGCGCAAGCCCGGAATCG |
| 9-2-DGK1-PAM2-F | AAAGGTCTCTCGTAAGGTGTTCCATTCTTCGTTTTAGAGCTAGAAATAGCAAGTTAAAATAAGGC |
| 9-2-DGK1-PAM2-R | TTTGGTCTCTACCATGCGCAAGCCCGGAATCG |
| 9-3-DGK1-PAM3-F | AAAGGTCTCTTGGTCATTTAACACCCAAAGGTTTTAGAGCTAGAAATAGCAAGTTAAAATAAGGC |
| 9-3-DGK1-PAM3-R | TTTGGTCTCTCATATGCGCAAGCCCGGAATCG |
| 9-4-DGK1-PAM4-F | AAAGGTCTCTTATGCTTTACTGTAGAACTGGTTTTAGAGCTAGAAATAGCAAGTTAAAATAAGGC |
| 9-4-DGK1-PAM4-R | TTTGGTCTCTAAAATAAATTGGCCATAGAAAAATTCTGTTATCCAC |
| 10-2-PAH1-PAM1-R | TTTGGTCTCTCACGTGCGCAAGCCCGGAATCG |
| 10-3-PAH1-PAM1-F | AAAGGTCTCTCGTGGAATCAGCTTGTCCAGGTTTTAGAGCTAGAAATAGCAAGTTAAAATAAGGC |
| 10-3-PAH1-PAM1-R | TTTGGTCTCTACCGTGCGCAAGCCCGGAATCG |
| 10-4-PAH1-PAM1-F | AAAGGTCTCTCGGTTCCTCCTTCTAAGATGGTTTTAGAGCTAGAAATAGCAAGTTAAAATAAGGC |
| PAH1-UP-F | GTTTCGTGTGCGTATAGAAAAGTATTTACC |
| PAH1-UP-R | ATTCTAAAGCCATCTCAAAAGCAAAAAGCTCTCTGTGTCAAAGGC |
| PAH1-DOWN-F | GAGAGCTTTTTGCTTTTGAGATGGCTTTAGAATTTGAAATTTGC |
| PAH1-DOWN-R | TTCCCTCCCTTTAGACAATGGGC |
| DGK1-UP-F | CTAGATTTAGATCTATTCACATCTTGAAGGTTGC |
| DGK1-UP-R | CCTGTTTTTGGTATATATGCTTATTGTCTGTAAACCCTGTATGAATCCAAATG |
| DGK1-DOWN-F | GGGTTTACAGACAATAAGCATATATACCAAAAACAGGAGTGTTTTATATTCTTC |
| DGK1-DOWN-R | ATCGCAGATAAACCATATATCCTTGAGATGC |

**Table S2 the primers of multi-copy sites integration**

| **Oligonucleotides** | **Sequences, 5'-3'** |
| --- | --- |
| GAL7-F-TDS | CAAGACAAACATATGCACTCTATGATTTGCCAGCTTACTATCCTTCTTGA |
| GAL7-R-ERG2 | TTTTGAGGGAATATTCAACTGTTTTTTTTTATCATGTTG |
| ERG2-F-GAL7 | CAGTTGAATATTCCCTCAAAAATGAAGTTTTTCCCACTCCTTTTGTTGATTG |
| ERG2-R-TEFT | CGGCCGGTACCTTAGAACTTTTTGTTTTGCAACAAGTTCTTACCC |
| TEFT-F-ERG2 | GTTGCAAAACAAAAAGTTCTAAGGTACCGGCCGCAAATTAAAGC |
| TEFT-R-ERG3 | GAAGAACAACTGACTCGAGTCATGTAATTAGTTATGTCACGC |
| ERG3-F-TEFT | ACTAATTACATGACTCGAGTCAGTTGTTCTTCTTGGTATTTGGGTCG |
| ERG3-GAL10P-R | CGTCAAGGAGAAAAAACTATAATGGATTTGGTCTTAGAAGTCGCTG |
| GAL10P-F-ERG3 | GACCAAATCCATTATAGTTTTTTCTCCTTGACGTTAAAGTATAGAGGTATATTAAC |
| GAL10P-R-CTT1 | CTTATTGGCATTTATATTGAATTTTCAAAAATTCTTACTTTTTTTTTGGATGG |
| CTT1-F-GAL10P | GTAAGAATTTTTGAAAATTCAATATAAATGCCAATAAGATCAATCAGCTCAGC |
| CTT1-R-TER22 | CAACATGGAAGCCAACCTATTTAATTGGCACTTGCAATGGACC |
| TER22-F-CTT1 | CTTGGTCCATTGCAAGTGCCAATTAAATAGGTTGGCTTCCATGTTGGC |
| TER22-R-DHCR24 | ATAAAATTTGTAAAGCAGCAAGACATTAAAATGTCAAAAGCCTCAAGGTGCC |
| DHCR24-F-TER22 | CTTTTGACATTTTAATGTCTTGCTGCTTTACAAATTTTATCATAAACTTC |
| DHCR24-R-TDH1P | CACTAAATTTACACACAAAACAAAATGTCTGCTGTTTGGTCTTTAGGTG |
| TDH1P-F-DHCR24 | CAGCAGACATTTTGTTTTGTGTGTAAATTTAGTGAAGTACTGTTTTTTG |
| TDH1P-R-TTI | GGATGGCGTATGAGTGGAAACCACACCGTGGGGCCTT |
| TTI-F-TDH1P | GGCCCCACGGTGTGGTTTCCACTCATACGCCATCCTTAAAGACCTG |
| TDS-R-GAL7 | GATAGTAAGCTGGCAAATCATAGAGTGCATATGTTTGTCTTGATAGG |
| THMG1-F | TTAGGATTTAATGCAGGTGACGGACCC |
| THMG1-R | TTTTCACCAATTGGTCTGCAGCCAT |
| IDI1-F | ATGACTGCCGACAACAATAGTATGC |
| IDI1-R | GAAAATGACAGGCAAATTCATAGAATGCTATAA |
| GAL1/10P-F | TATAGTTTTTTCTCCTTGACGTTAAAGTATAGAGGTATATTAAC |
| GAL1/10P-R | TTATATTGAATTTTCAAAAATTCTTACTTTTTTTTTGGATGG |
| TY12-DOWN-F | TAAAACGGAATGATGAATAATATTTATAGAATTGTGTAG |
| TY12-DOWN-R | CACTCAGACCTGAAGTGAAGTTCCTATAC |
| TY12-UP-F | CCGCGCTGAGGGTTTAATGGCG |
| TY12-UP-R | GCAAGGATTGATAATGTAATAGGATCAATGAATATTAACATA |
| TY2-UP-F | GTGTCCGCGCTGAGGGTTTAATG |
| TY2-DOWN-R | GTATAGGAACTTCACTTCAGGTCTGAGTG |

**Table S3 the yeast used in this study**

| **Name** | **Description** | **Source** |
| --- | --- | --- |
| C800 | CENPK2-1D; *MATα*, *ura3-52*, *trp1-289*, *leu2-3*,*112*, *his3Δ1*, *MAL2-8^C^*, *SUC2*, *gal80*::*KanMX* | (Gao et al., 2020) |
| *7-DHC-1* | C800; Δ*ERG5*:: P*_TEF1_*-*DHCR24*-T*_TEF1_*，Δ*ERG6*:: P*_TEF1_*-*DHCR24*-T*_TEF1_* | This study |
| *7-DHC-2* | *7-DHC-1*; Δ*MOT3*:: P*_PGK1_*-*ERG2*-T*_TEF1_* ，P*_TDH3_*-*ERG3*-T*_TEF1_* | This study |
| *7-DHC-3* | *7-DHC-2*; Δ*NEM1* | This study |
| *7-DHC-4* | *7-DHC-3*; Δ*Ty1*:: P*_GAL1_*- *tHMG1*-P*_GAL10_*- *IDI1* - *SpHIS5*deg | This study |
| *7-DHC-5* | *7-DHC-3*; Δ*Ty2*:: P*_GAL7_*-*ERG2*-P*_GAL1_*-*ERG3*- P*_GAL10_*-*CTT1*-P*_TDH1_*-*DHCR24*- *KlURA3*deg | This study |
| *7-DHC-6* | 7-DHC-5; Δ*Ty1*:: P*_GAL1_*- *tHMG1*-P*_GAL10_*- *IDI1* - *SpHIS5*deg | This study |
| *7-DHC-7* | 7-DHC-6; Δ*Ty3*:: P*_GAL7_*-*ERG2*-P*_TDH3_*-*ERG3*- P*_PGK1_*-*POS5*-P*_TDH1_*-*DHCR24*- *ScTRP1*_AAG_^Anti^deg | This study |

Gao, S., Xu, X.Y., Zeng, W.Z., Xu, S., Lyv, Y.B., Feng, Y., et al. (2020). Efficient biosynthesis of (2S)-eriodictyol from (2S)-naringenin in *Saccharomyces cerevisiae* through a combination of promoter adjustment and directed evolution. *ACS Synth.Biol.* 9(12)**,** 3288-3297. doi: 10.1021/acssynbio.0c00346.
